# A Giant Coronary Artery Aneurysm with Coronary Arteriovenous Fistula in Asymptomatic Elderly Patient

**DOI:** 10.1155/2013/847972

**Published:** 2013-11-12

**Authors:** Caterina Milici, Daniella Bovelli, Valentino Borghetti, Georgette Khoury, Marco Bazzucchi, Massimo Principi, Marcello Dominici, Enrico Boschetti

**Affiliations:** ^1^Cardio-Thoracic and Vascular Department, University Hospital of Terni, Via Tristano di Joannuccio 1, 05100 Terni, Italy; ^2^Radiology Department, University Hospital of Terni, Via Tristano di Joannuccio 1, 05100 Terni, Italy

## Abstract

Coronary Arteriovenous Fistula (CAF) is a rare defect that occurs in 0.1-0.2% of patients undergoing coronary angiography; Coronary Artery Aneurism (CAA) also occurs in approximately 15–19% of patients with CAF. It is usually congenital, but in rare occasions it occurs after chest trauma, cardiac surgery, or coronary interventions. The case described is that of a 72-year-old woman, without previous history of cardiovascular disease, who presented a huge cardiac mass. A multimodal approach was necessary to diagnose a giant CAA with CAF responsible for compression and displacement of cardiac structures. Due to likely congenitally origin of the lesion and the absence of symptoms correlated to the CAA and to the CAF we decided to avoid invasive interventions and to treat the patient with medical therapy.

## 1. Case Report

A-72-years old white woman was admitted to our department for recently sudden onset of dyspnea, asthenia, and profuse sweating. At physical examination there were normal hemodynamic parameters (BP 120/80 mmHg, HR 95 bpm), normal SaO_2_ (97%), and body temperature 36°C. The electrocardiogram (ECG) showed sinus rhythm with morphologically infero-lateral aspecific modification of ventricular repolarization. A wide mediastinal shadow was evidenced at chest X-ray; therefore, the patient was submitted to transthoracic echocardiogram (TTE) which detected an intrapericardial, capsulated mass (max diameter 9 cm) associated with a pericardial effusion (See Supplementary Movie 1 and Movie 2 in Supplementary Material available inline at http://dx.doi.org/10.1155/2013/847972). A better diagnostic mass definition was completed by Transesophageal Echocardiogram (TEE) which showed a calcified regular profiled capsule with concentric areas of a different echogenicity apparently in contiguity with the inferior part of the interatrial septum (Movie 3). The body mass was vacuolated inside with central swirling slow flow. No hemodynamic alteration was induced by the mass due to compression or dislocation of surrounding cardiac structures. Both of the two atria were morphologically normal except for a patent oval foramen. Coronary sinus was not clearly detectable. Both ventricles showed normal dimensions and function. 

**Figure 1:**
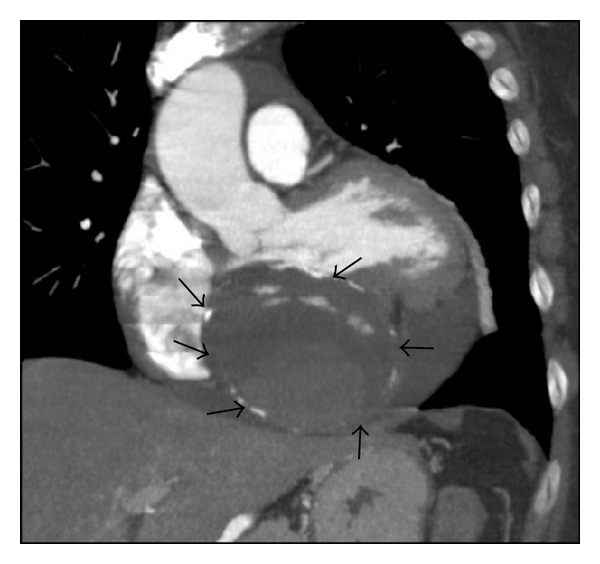
Multidetector Computed Tomography (MDCT) multiplanar reconstruction (MPR) image on oblique coronal plane demonstrates an intrapericardial grossly round mass (arrows) between the left atrium and the left ventricle. The mass impresses the posterior wall of the left ventricle and the atrium. In this early arterial phase the center of the mass is not yet opacified. Pericardial effusion coexists and surrounds the mass.

**Figure 2:**
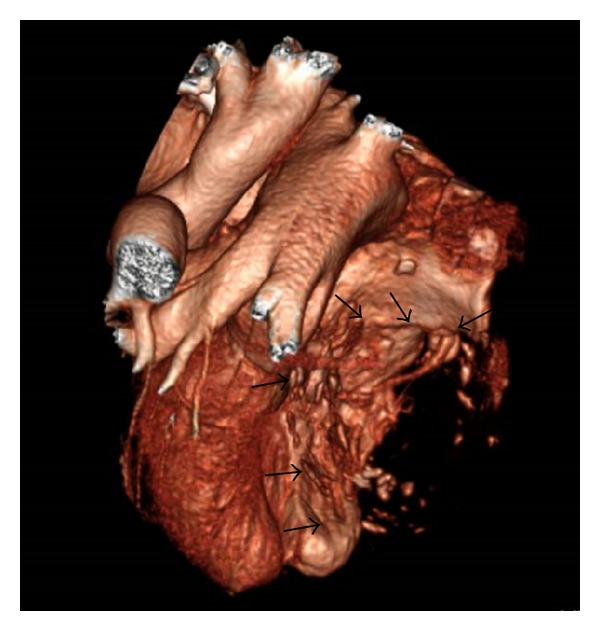
Volume rendering Computed Tomography (CT) image, obtained by the exclusion of the mass (arrows), better demonstrates the effect of the lesion on the heart, in particular the markedly impressed and dislocated cardiac walls.

A 16-sliced contrast-enhanced Multidetector Computed Tomography (MDCT) allowed identifying the extracardiac location of the giant capsulated mass, placed between the left ventricle and left atrium. Anatomical structure was composed of a camera of two layers of stratified calcific shells internally coated by a thick organized thrombus containing a central area of hematic flow (Figure 1). Right atrium was morphologically grossly altered due to its extracardiac compression. Proximal right coronary artery appeared ectasic and tortuous but the crux cordis and its distal part were not detectable. Owing to slow hematic flow, the cardiac mass was evidenced only during a late contrast phase. The giant dimension of the cardiac mass was responsible of cardiac structures displacement and difficult definition of venous coronary drainage system (Figure 2).

**Figure 3:**
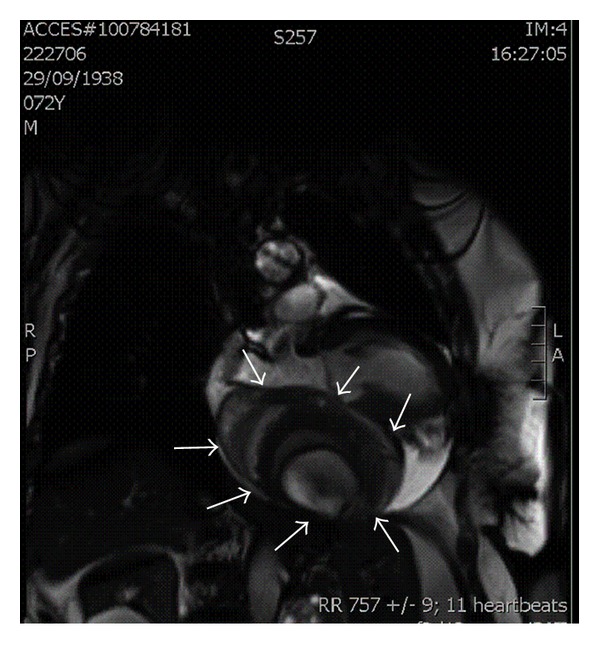
3-Tesla Cardiac Magnetic Resonance-Steady State Free Precession (SSFP) image on vertical long axis of the left ventricle: a giant capsulated intrapericardial mass is detectable (arrows), placed between the left ventricle and left atrium, with stratified calcific shells inside and an internal layer of a thick organized thrombus containing a central area of turbulent blood flow. Right atrium is morphologically grossly altered due to the extracardiac deformation.

3-Tesla Cardiac Magnetic Resonance Imaging (MRI) was then performed (Figure 3), without adding any further diagnostic elements.

**Figure 4:**
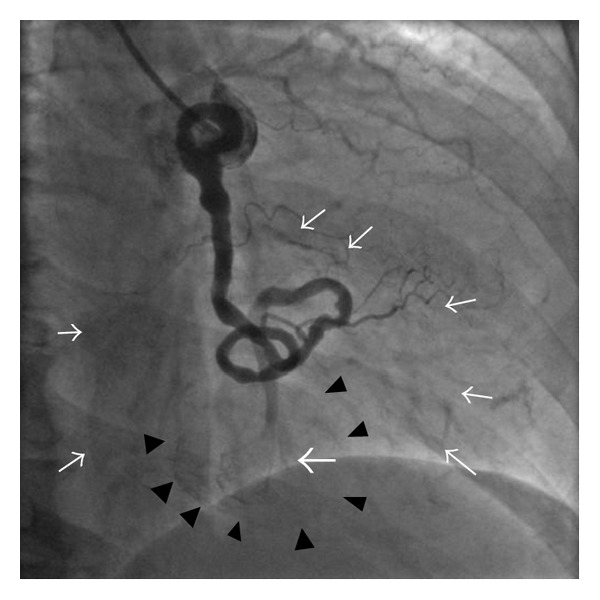
Coronary artery angiography (CAG) in right anterior oblique (RAO) 30° view. Blood flow from Right Coronary Artery (arrow) drains into the cavity (arrow heads) of the mass (white arrows).

The patient was therefore submitted to coronary artery angiography (CAG). Left coronary artery (LCA) was normal whereas the right coronary artery (RCA) was tortuous and ectasic with its anatomic course ending in a bulky cavity. (Movie 4 and Figure 4).

A giant coronary aneurysm of the peripheral segment of RCA was diagnosed and, although neither MDCT nor CAG detected any drainage vessel, a coronary artery fistula (CAF) draining into the coronary sinus seemed to be the most likely diagnosis.

Moreover, a dynamic contrast-Transthoracic Echocardiogram (SonoVue-Bracco International) showed a microbubbles diastolic flow inside the vacuolated mass without any communication with the pericardium therefore supporting the diagnosis of right coronary to coronary sinus fistula (Movie 5).

Due to likely congenitally origin of the lesion and the absence of symptoms correlated to the Coronary Artery Aneurysm (CAA) and to the CAF, patient was discharged with medical therapy and referred for clinical followup.

## 2. Discussion

CAF is a rare defect that occurs in 0.1-0.2% of patients undergoing coronary angiography. It is usually congenital but in rare occasions is acquired after chest trauma, cardiac surgery, or coronary interventions. Vascular anomaly may involve any portion of coronary tree but in approximately 55% of the cases it originates from right coronary artery, 40% from the left main coronary artery, and 5% from both. The usual drainage sites are the right ventricle in 41%, right atrium in 26%, pulmonary artery in 17%, coronary sinus in 7%, left ventricle in 3%, and superior vena cava in 1% of cases. A left-to-right shunt exists in more than 90% cases. CAA also occurs in approximately 15–19% of patients with CAF [1]. The reason for this behavior is that a fistula consists of a “nest” of vessels containing fragile smooth muscle, which is susceptible to constant exposure to arterial pressure and much blood flow, resulting in dilatation and aneurysmal change with age [1, 2].

In our case the patient had no history of chest trauma, cardiac surgery, or coronary intervention, and hence we concluded that she had congenital CAF and developed a giant CAA.

We thought that the fistula may be formed by direct erosion and rupture of the aneurysm into the coronary sinus due to progressive atherosclerosis resulted from long-term exposure to arterial pressure, with evidence of calcified walls of the aneurysm, as observed by Makaryus et al. in a similar case [3]. This was suggested by the presence of stratified calcific shells inside and an internal layer of a thick organized thrombus containing a central area of turbulent blood flow, as shown by 3-Tesla Cardiac Magnetic Resonance (Figure 3).

Despite CAA is currently diagnosed with noninvasive tools (TTE, TEE, or magnetic resonance), CAG is the gold standard because it provides information on the location of CAA and fistula origin. However, sometimes CAG is limited in its ability to determine the course and drainage site of fistulas due to the overlap of adjacent structures.

In our case a multimodal approach was necessary because of the unusual giant CAA which was responsible of compression and displacement of cardiac structures causing a macroscopically heart anatomy derangement.

Clinical symptoms and age at manifestation of a congenital CAF depend on the underlying anatomy and on the size of the fistulous connection to the left or right side of the heart. The most frequent symptoms and fistula-related complications are dyspnea on exertion, palpitations, congestive heart failure, infective endocarditis, and death [4]. 

Since the natural course of CAA with CAF is unclear, the management of these patients is controversial [2].

Most studies have found that symptoms and complications increase with age making surgical correction recommended before the development of symptoms and when left to right shunt (*Q*_*p*_/*Q*_*s*_) is >1 : 1.5 in asymptomatic patients [5]. Current treatment options for CAF include fistula surgical ligation and transcatheter closure. In our case we did not consider transcatheter closure of this CAF because the exact angiographic visualization of the anatomy was not possible and the huge dimension limited any invasive treatment since unsuccessful attempts in patients with giant aneurysms have been reported in the literature.

When surgery is suggested, the exact angiographic visualization of the CAF anatomy and the regular coronary vessels branching off proximally and distally of the fistula is of paramount importance [5].

Our case is one of the largest asymptomatic CAA with CAF ever reported to our knowledge.

Due to congenital origin of the abnormality, the elderly age, the absence of symptoms, and the difficulty to recognize exactly draining vessel, we decided to treat this patient with medical therapy planning a watchful waiting followup.

## Supplementary Material

Movie 1: Transthoracic Echocardiogram (TTE), parasternal long-axis view. An intrapericardial apparently capsulated cardiac mass (max diameter 9cm) is detectable at the level of left atrial region; echogenicity inside the mass is quite irregular, with a thicker core.
Movie 2: Transthoracic Echocardiogram (TTE), subcostal view. The mass is detectable at interatrial septum level, but it doesn't seem to be related to any cardiac structure.
Movie 3: Transesophageal echocardiogram (TEE). This exam allows a better definition of the mass, which shows a calcified regular profiled capsule and concentric areas of different echogenicity inside, with a central swirling slow flow; apparently it is in contiguity with the inferior part of the interatrial septum.
Movie 4: Coronary artery angiography (CAG) in right anterior oblique (RAO) 30° view showing a tortuous and ectasic right coronary artery with its anatomic course ending in a bulky cavity.
Movie 5: Contrast-Transthoracic Echocardiogram, performed with SonoVue (Bracco International) showing a microbubbles flow inside the vacuolated camera of the cardiac mass without any communication with pericardial effusion.
